# Epidemiology of *Clostridioides difficile* Infection in Argentina and Associated Risk Factors Evaluated Through a Meta-Analysis

**DOI:** 10.3390/antibiotics15060528

**Published:** 2026-05-22

**Authors:** Angela María Barbero, Nicolás Diego Moriconi, Sabina Palma, Josefina Celano, María Gracia Balbi, Lorenzo Sebastián Morro, María Martina Calvo Zarlenga, Jorgelina Suárez, María Guadalupe Martínez, Mónica Graciela Machain, Carlos Gabriel Altamiranda, Gabriel Erbiti, Rodrigo Emanuel Hernández Del Pino, Virginia Pasquinelli

**Affiliations:** 1 Centro de Investigaciones Básicas y Aplicadas (CIBA), Universidad Nacional del Noroeste de la Provincia de Buenos Aires (UNNOBA), Newbery 261, Junín 6000, Buenos Aires, Argentina; ambarbero@comunidad.unnoba.edu.ar (A.M.B.); ndmoriconi@comunidad.unnoba.edu.ar (N.D.M.); sabinapalma.sp@gmail.com (S.P.); rhernandezdp@gmail.com (R.E.H.D.P.); 2 Centro de Investigaciones y Transferencias del Noroeste de la Provincia de Buenos Aires (CIT NOBA), UNNOBA-Universidad Nacional de San Antonio de Areco (UNSAdA)-Consejo Nacional de Investigaciones Científicas y Técnicas (CONICET), Monteagudo 2772, Pergamino 2700, Buenos Aires, Argentina; 3 Universidad Nacional de San Antonio de Areco (UNSAdA), Zapiola 362, San Antonio de Areco 2760, Buenos Aires, Argentina; 4 Hospital Interzonal General de Agudos (HIGA) Dr. Abraham F. Piñeyro, Lavalle 1084, Junín 6000, Buenos Aires, Argentina; 5 Clínica Centro, Presidente Quintana 249, Junín 6000, Buenos Aires, Argentina; 6 Departamento de Humanidades, Universidad Nacional del Noroeste de la Provincia de Buenos Aires (UNNOBA), Libertad 555, Junín 6000, Buenos Aires, Argentina

**Keywords:** *C. difficile*, epidemiology, risk factors, meta-analysis

## Abstract

Background: *Clostridioides difficile* is classified within the first 18 threats for antimicrobial resistance and is the leading cause of hospital-acquired enteric infection. Community-associated cases have notably increased in recent decades, highlighting that accurate and up-to-date statistics characterizing the epidemiology of *C. difficile* infection (CDI) are critical. Methods: We conducted a retrospective (2019–2023) case-control study evaluating the prevalence of CDI in 249 stool samples from hospitalized patients in the sanitary region III of Buenos Aires, Argentina. The presence of *C. difficile* was detected by combining EIA, PCR, and toxigenic culture via a diagnostic algorithm. Clinical and demographic data from patients were analyzed to identify CDI-associated risk factors. We also conducted a systematic review and a meta-analysis contrasting our results with 38 studies selected from different countries. Results: One in five patients presented *C. difficile* as the etiological agent of diarrhea. Eighty percent of the CDI+ cases carried toxigenic strains, with a third of cases associated with community environments. Age ≥ 69 years, previous use of antibiotics, previous hospitalization, and previous episodes of CDI emerged as predisposing factors for CDI in our study cohort. In an exploratory evaluation of clinical data, CDI+ patients showed higher leukocytes and platelets counts, a decreased basophil count, and increased urea concentration. At the global level, the meta-analysis reinforced advanced age, previous consumption of antibiotics, previous consumption of proton pump inhibitors, previous hospitalization, and previous CDI as risk factors for CDI. Conclusions: These results emphasize the importance of continued epidemiological surveillance of CDI. Our findings confirm previously described risk factors, both in our cohort and at the global level. Exploratory alterations in laboratory parameters were observed, although their clinical relevance and specificity require further investigation.

## 1. Introduction

*Clostridioides difficile* infection (CDI) is considered the most common hospital-acquired disease [1,2]. Since 2019, the Centers for Disease Control (CDC) have identified *C. difficile* as an “urgent threat”, with the immediate need to implement prevention and control actions [3]. *C. difficile* is a Gram-positive, anaerobic, and spore-forming bacterium that causes intestinal damage, primarily through toxin A (TcdA) and toxin B (TcdB) [4]. CDI can be life-threatening, ranging from mild diarrhea to pseudomembranous colitis and causing nearly 500,000 cases per year in the United States [5]. The main transmission route for *C. difficile* is through direct person-to-person contact via the fecal–oral route. *C. difficile* spores constitute the main form of resistance and can persist in the environment for long periods [6]. Additionally, asymptomatic carriers shed bacteria in their feces, contributing to environmental contamination and transmission to other people [7].

CDI is also a major economic burden on healthcare systems and is closely linked to the high recurrence rates of this infection. Although standard treatments resolve CDI in most cases, up to 35% of treated patients experience disease recurrence with aggravated symptoms [5]. Among these patients, up to 60% may experience additional recurrences, demonstrating a pattern of multiple episodes [5]. Therapies for CDI include antibiotics such as metronidazole for mild cases and vancomycin or fidaxomicin for moderate to severe cases, while more specialized strategies like anti-toxin antibodies or Fecal Microbiota Transplant (FMT) are recommended for recurrences [8].

With respect to global epidemiology, while healthcare-associated CDI has declined in recent years, the incidence of community-associated CDI is on the rise [9,10,11,12,13,14]. In developing countries, patients with diarrhea are not routinely screened for *C. difficile*, which leads to an underdiagnosis and places a significant economic burden on the healthcare system. Attempts to follow international guidelines for CDI diagnosis have been made in Latin American countries; however, the high cost of the methods imposes the use of the most cost-effective tests based on local conditions [15]. Although CDI is increasingly recognized as a global public health concern, epidemiological evidence from Latin America, and especially from Argentina, is still scarce and heterogeneous.

While there are currently several risk factors associated with the development of CDI, a thorough and up-to-date understanding of the features and predisposing conditions is essential for the development of effective prevention and management strategies. The use of antimicrobials, with an emphasis on broad-spectrum antibiotics, has been described as the most significant risk factor for CDI [16,17]. These antibiotics can disrupt the normal balance of bacteria in the gastrointestinal tract, generating dysbiosis and allowing *C. difficile* to overgrow in the gut. Hospitalization in healthcare facilities, especially those with prolonged stays, is another well-characterized risk factor for CDI [17]. Advanced age, which can be related to a weakened immune system and a greater likelihood of residing in healthcare settings, is also an important predisposing condition [17]. The use of stomach acid regulators, such as proton pump inhibitors (PPIs) and H2 blockers, has been associated with an increased risk of CDI [18]; however, this is still under debate. Previous episodes of CDI heighten the risk of recurrence [19]. Finally, several underlying health conditions that could compromise the immune system could contribute to augmented susceptibility or severity of the infection. Comorbidities such as inflammatory bowel disease (IBD) [20], Crohn’s disease [20], ulcerative colitis [20], diabetes [21], and chronic kidney disease (CKD) [22] can increase the risk of CDI. Certain clinical procedures (e.g., chemotherapy and gastrointestinal surgery), malnutrition or enteral nutrition, organ transplantation along with immunosuppressive medications, and blood disorders may also increase the risk of CDI [23,24]. Here, we conducted a systematic review and meta-analysis of commonly reported factors that are associated with the development of CDI to comprehensively evaluate the global trends of risk conditions.

In this retrospective study, we were not only able to assess the prevalence of *C. difficile* in the northwest region of Buenos Aires, Argentina, but also to integrate our findings with a meta-analysis, providing an update and a deeper characterization of risk factors reported globally.

## 2. Results

### 2.1. CDI Prevalence

In Argentina, studies and reports on CDI are scarce and heterogeneous [25,26,27,28,29,30,31,32]. To contribute to a better understanding of the disease prevalence, we conducted a retrospective analysis of 249 fecal samples received between 2019 and 2023 from Sanitary Region III of Buenos Aires (Supplementary Figure S1a,b).

**Figure 1:**
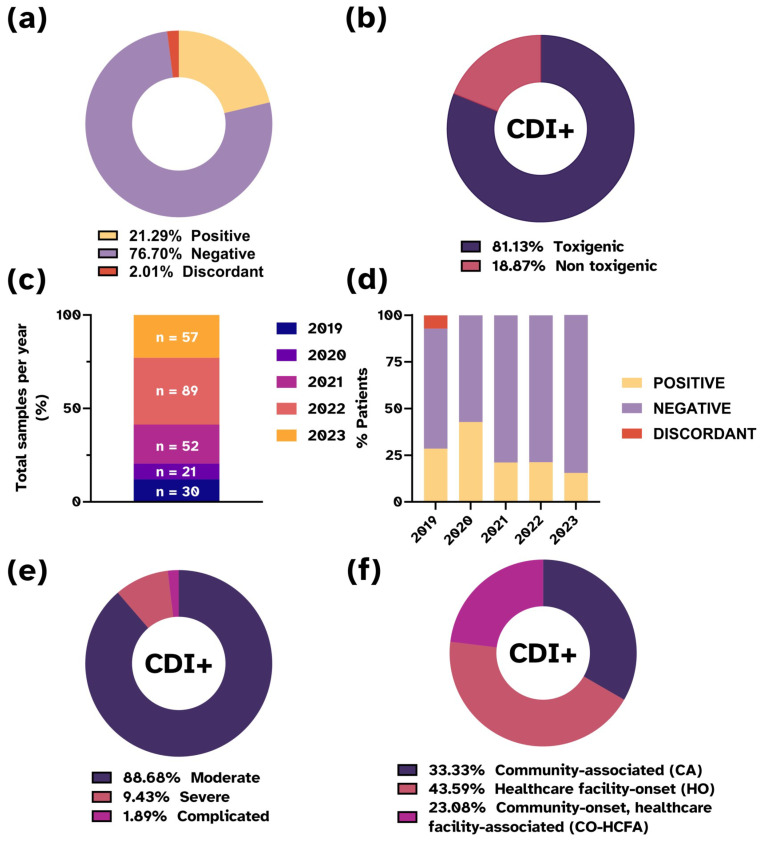
CDI prevalence. A total of 249 stool samples were evaluated via a diagnostic algorithm that includes Enzyme Immunoassay (EIA), direct PCR of stool samples, and toxigenic culture (plate culture followed by PCR). All three tests allow the detection of glutamate dehydrogenase (GDH) and *C. difficile* Toxin B. (**a**) Donut graph showing positive, negative, and discordant (only positive for PCR or toxigenic culture when the other two tests were negative) results for the presence of *C. difficile*. (**b**) Donut graph showing the percentage of toxigenic strains (*C. difficile* containing Toxin B) detected in the stool samples from CDI+ patients. (**c**) Percentage of total cases (CDI− and CDI+) analyzed per year between 2019 and 2023. (**d**) Percentage of CDI+, CDI−, and discordant results classified on an annual basis from 2019 to 2023. (**e**) CDI severity classification following the criteria established by the Infectious Diseases Society of America (IDSA). (**f**) CDI classification by setting of acquisition.

We identified *C. difficile* as the causal agent of one of the five diarrhea cases (21.29%) (Figure 1a). More than 80% of the patients were infected with toxigenic strains, indicated by the presence of *C. difficile* Toxin B in the stool samples (Figure 1b).

When the cases were analyzed on an annual basis (Figure 1c,d), the frequency of CDI ranged from 15.52 to 42.86% from 2019 to 2023 (Figure 1d). Interestingly, the highest prevalence in our study cohort was observed in 2020, which was a duplicate of the percentage of CDI+ cases (42.86%) compared with the other years (mean = 21.65%, Figure 1d).

Most patients presented moderate CDI severity (Figure 1e). Notably, the majority of CDI infections (56.41%) had a community onset (CA + CO-HCFA) (Figure 1f). Regarding the setting of acquisition, 33.33% of the patients presented with community-associated CDI (CA), 23.08% with community CDI associated with healthcare environments (CO-HCFA), and the remaining 43.59% of the patients were classified as healthcare facility-associated (HO) cases (Figure 1f).

### 2.2. Risk Factors

After classifying the patients as CDI+ or CDI−, we determined the risk factors that could be involved in the prevalence of CDI.

**Figure 2:**
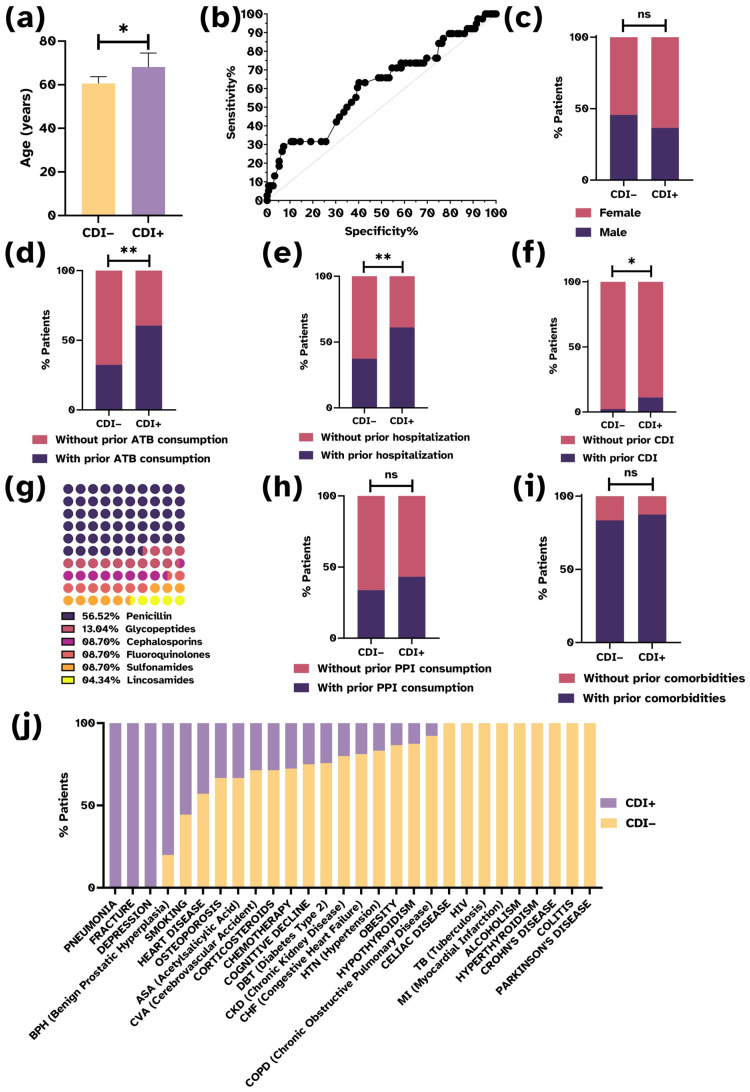
CDI-associated risk factors. (**a**) Age (years), (**b**) ROC curve to establish the age cut-off point, (**c**) sex assigned at birth, (**d**) antibiotic (ATB) consumption in the 3 months prior to hospitalization, (**e**) prior hospitalization in the last 28 days, (**f**) prior CDI, (**g**) antibiotic breakdown by family, (**h**) PPI consumption prior to hospitalization, (**i**) comorbidities, (**j**) breakdown of specific comorbidities. (**a**), Mann–Whitney test, and (**c**–**i**), Fisher’s exact test. Stacked bars represent the percentage of patients for each parameter. ns = non-significant; * *p* < 0.05; ** *p* < 0.01.

We found significant differences in the age of the CDI+ and CDI− populations, with the CDI+ patients presenting a greater average age (Figure 2a, CDI+: mean = 68.16 years vs. CDI−: mean = 60.61 years). By using ROC curve analysis, we established a cut-off point of 69.50 years for advanced age as a risk factor in our cohort (Figure 2b). With respect to sex assigned at birth, no differences were found in the proportions of males and females between the patient populations (Figure 2c).

The consumption of antibiotics within the 3 months prior to the diagnosis of CDI, as well as previous hospitalizations and infections with *C. difficile*, could be considered risk factors for CDI in our study cohort (Figure 2d–f). When the families of antibiotics consumed by CDI+ patients were analyzed, we observed that 56.52% had taken some kind of penicillin prior to diagnosis (Figure 2g).

On the other hand, the consumption of proton pump inhibitors (PPIs) prior to diagnosis and the presence of comorbidities did not significantly differ between CDI− and CDI+ patients (Figure 2h,i). Figure 2j shows a breakdown of the comorbidities reported in the patients under study. Although they were analyzed individually, no substantial variations were found between the CDI+ and CDI− populations for any of them.

No differences were found in the variables related to the evolution of patients during admission to health centers, such as hospitalization in common floors or ICUs, need for an ICU, or shock or death (Supplementary Figure S2).

### 2.3. Blood and Serum Parameters

We also analyzed the patients’ blood counts and serum parameters, which were measured on the day of fecal sample collection.

**Figure 3:**
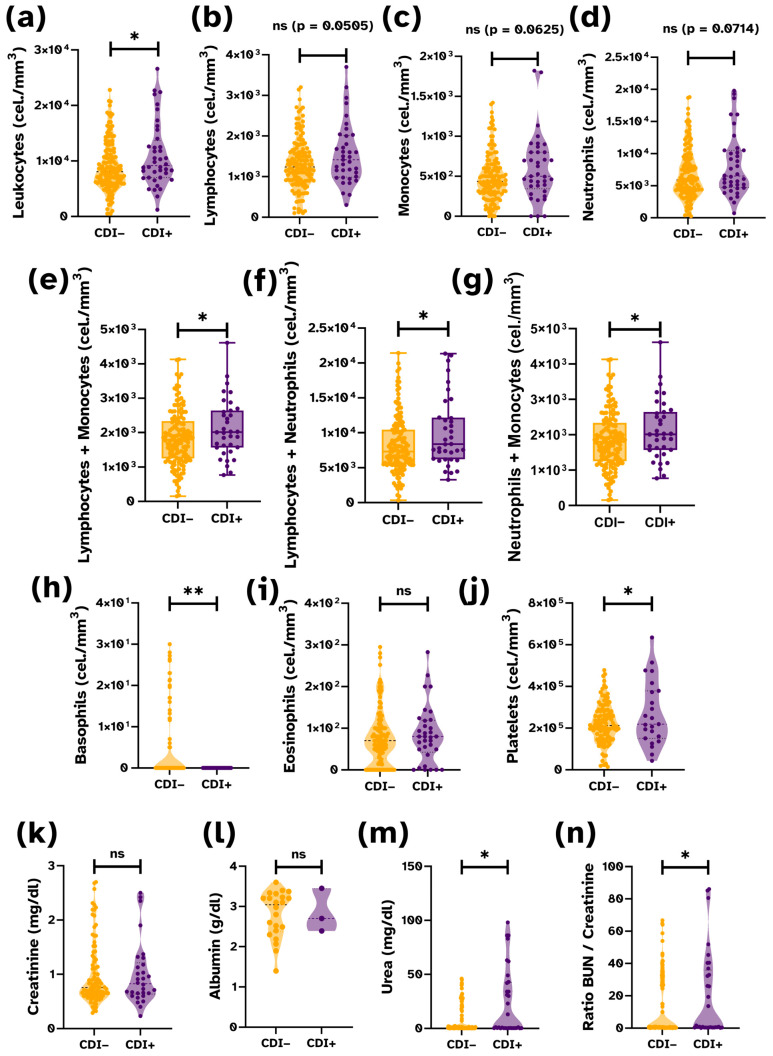
Blood and serum parameters in CDI+ and CDI− patients. Blood samples from CDI+ and CDI− patients were obtained on the same day as fecal sample collection. The number of (**a**) leukocytes (cells/mm^3^), (**b**) lymphocytes (cells/mm^3^), (**c**) monocytes (cells/mm^3^), (**d**) neutrophils (cells/mm^3^), (**e**) lymphocytes plus monocytes (cells/mm^3^), (**f**) lymphocytes plus neutrophils (cells/mm^3^), (**g**) neutrophils plus monocytes (cells/mm^3^), (**h**) basophils (cells/mm^3^), (**i**) eosinophils (cells/mm^3^), and (**j**) platelets (cells/mm^3^) were quantified in a hematology analyzer. Levels of (**k**) creatinine (mg/dL), (**l**) albumin (g/dL), and (**m**) urea (mg/dL). (**n**) BUN (blood urea nitrogen)/creatinine ratio. (**a**–**i**) and (**k**–**n**) Mann–Whitney test. (**j**), unpaired *t*-test. Violin plots show the distribution of the data. ns = non-significant; * *p* < 0.05; ** *p* < 0.01.

We observed a significant increase in the number of leukocytes (Figure 3a) and platelets (Figure 3j) in patients infected with *C. difficile*. When the individual lymphocyte, monocyte, and neutrophil counts were analyzed, although an increase was evident, we did not find significant differences between the patient populations (Figure 3b–d). However, at least two of these leukocyte populations were increased in the CDI+ patients compared with the CDI− patients (Figure 3e–g).

With respect to the other parameters, while a significant decrease in the basophil count was observed (Figure 3h), the eosinophil count did not seem to be affected by the presence of *C. difficile* (Figure 3i).

The serum concentrations of creatinine, albumin, and urea were also evaluated. No differences were evident in the creatinine and albumin levels (Figure 3 k,l), but an elevated urea concentration was detected in patients infected with *C. difficile* (Figure 3m). Moreover, the BUN (Blood Urea Nitrogen)/Creatinine ratio was significantly elevated in CDI patients (Figure 3n).

### 2.4. Meta-Analysis

**Figure 4:**
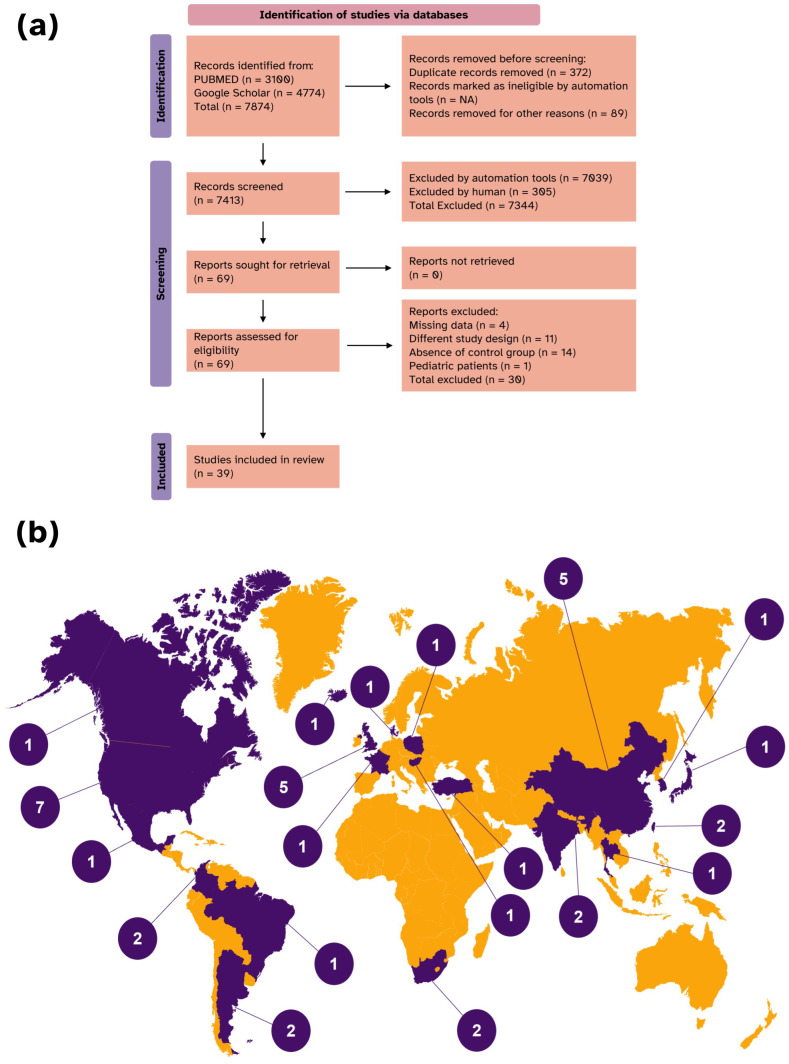
Flow chart of the studies and countries screened and included in the meta-analysis. (**a**) Identification and selection of the studies were performed following the PRISMA 2020 guidelines. After initial identification, eligibility criteria were applied, and 39 reports (included OUR STUDY) were selected for the meta-analysis. CDI− patients were considered the control group. (**b**) World map showing the countries from which the studies included in the meta-analysis were published (violet). The circles contain the number of studies selected per country.

The results obtained regarding the clinical and demographic characteristics of the CDI+ and CDI− patients in our cohort were compared with reports from other countries through a meta-analysis. We defined the selection criteria (Figure 4a) and carried out a systematic review that allowed us to select 38 independent case/control studies (Table 1) from the countries shown in violet in Figure 4b.

**Figure 5:**
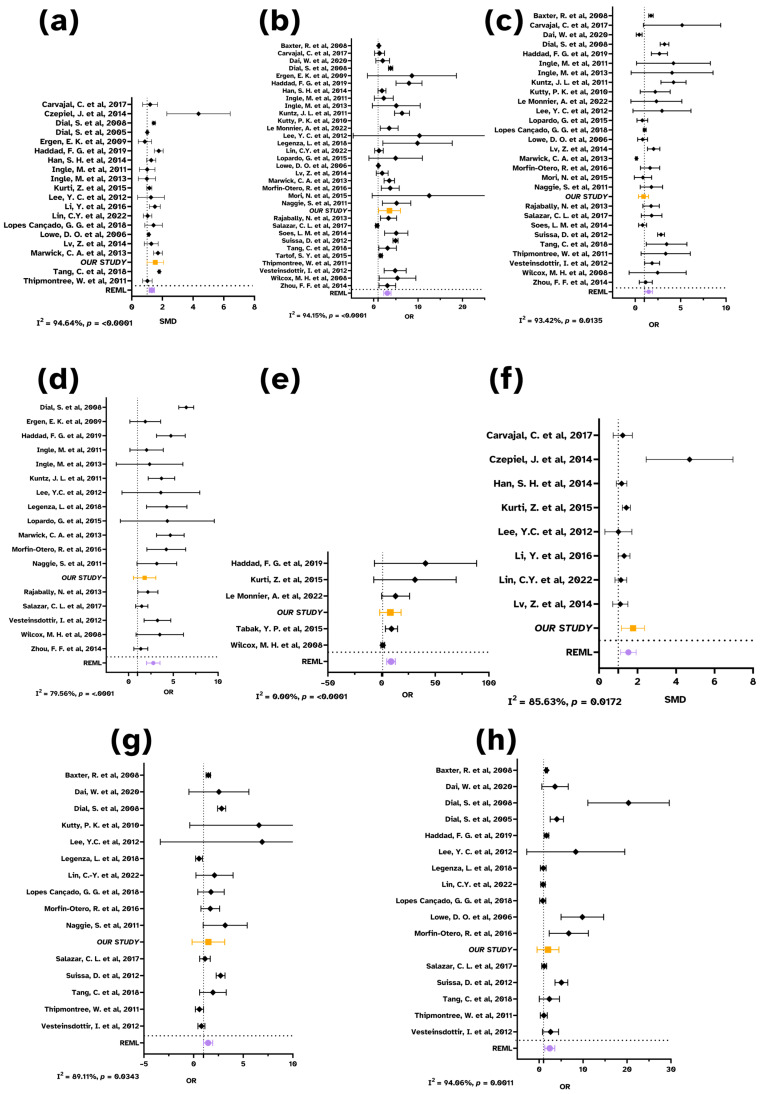
Forest plots of risk predictors. Representative forest plots of the meta-analysis. (**a**) Age, (**b**) ATB consumption in the previous 3 months, (**c**) PPI consumption in the previous 3 months, (**d**) prior hospitalization, (**e**) previous CDI, (**f**) white blood cell (WBC) count (cells/mm^3^), (**g**) heart disease, (**h**) chronic kidney disease. A REML (Random Effect Maximum Likelihood, light violet circle model) was applied. Models with *p* < 0.05 and OR/SMD ± CI values less than or greater than 1 were considered potential risk predictors for CDI. The black diamonds represent the means of each of the variables in each study. Our study (orange square) is mentioned as OUR STUDY. The purple circle in the forest plots represents the pooled overall effect size (Odds Ratio [OR] or Standardized Mean Difference [SMD]) calculated by the Random-Effects Maximum Likelihood (REML) model for the specific variable under analysis. (**a**) [33,34,36,37,38,40,44,46,47,50,53,54,55,56,57,58,59,60,64], (**b**) [27,34,35,37,38,39,40,41,42,43,45,46,47,48,49,51,52,53,54,55,56,57,59,60,61,62,63,64,65,66,67,69], (**c**) [27,33,34,35,37,38,39,40,41,42,43,45,46,47,48,49,52,54,56,59,60,61,62,63,64,65,66,67], (**d**) [27,39,41,45,46,47,49,51,52,54,57,59,60,62,64,65,67], (**e**) [43,44,62,64,68], (**f**) [36,37,40,44,50,53,54,55], (**g**) [33,35,38,41,45,49,51,54,55,56,59,61,63,66,67], (**h**) [33,34,35,38,41,45,49,51,54,55,56,58,59,61,63,64]. OR = odds ratio. SMD = standard media deviation. CI = confidence interval.

The forest plots obtained for each of the selected parameters of interest are shown in Figure 5 and Figure S3, and the effects for each of the variables evaluated in the meta-analysis are summarized in Table 2.

The dataset included 11,596 individuals with CDI and 536,467 matched controls (Table 3). Overall, the distribution of sex assigned at birth was 40.06% male vs. 59.94% female, with 44.33% vs. 55.67% in the CDI+ population and 39.98% vs. 60.02% in the control group. The demographic parameter age (OR = 1.30; 95% CI, 1.14 to 1.48) was significant, which implies that, globally, advanced age is associated with a greater risk of CDI (Figure 5a). On the other hand, there was no evidence on the risk of CDI for the sex assigned at birth (OR = 0.91; 95% CI, 0.30 to 0.99) (Supplementary Figure S3a).

A total of 9.11% of the included individuals had previously been exposed to antibiotics, and 22.18% had been exposed to PPIs. The consumption of antibiotics (CDI+ 39.95% vs. CDI− 8.52%) and PPIs (CDI+ 29.87% vs. CDI− 21.76%) was greater among patients infected with *C. difficile* (Table 3). In this analysis, which ignored antibiotic subclasses, we found that the pooled impact of any antibiotic exposure (OR = 3.02; 95% CI, 2.32 to 3.94) increased the risk of CDI by a factor of 3 (Figure 5b) while PPIs (OR = 1.43; 95% CI, 1.08 to 1.91) increased the risk of CDI by a factor of 1 (Figure 5c).

Previous hospitalization emerged as the third most influential predictor for CDI risk (OR = 2.69; 95% CI, 2.05 to 3.54, Figure 5d) after antibiotic consumption and previous CDI (Figure 5b,e). Among the CDI+ patients included in the meta-analyses, 27.88% had been hospitalized prior to diagnosis (Table 3), whereas only 16.80% had been hospitalized in the control group.

The previous CDI (OR = 8.20; 95% CI, 5.15 to 13.06) was the most influential predictor of risk (Figure 5e). When evaluating comorbidities, both heart diseases (OR = 1.41; 95% CI, 1.02 to 1.94) and chronic kidney disease (OR = 2.23; 95% CI, 1.38 to 3.61) exhibited significant effects (Figure 5g,h). Therefore, patients with these pathologies have an increased risk of CDI. The other tested comorbidities, diabetes *mellitus* and HIV, were not associated with a higher risk of infection (Table 2 and Supplementary Figure S3c,d).

Finally, blood parameters were analyzed. An increased leukocyte count is also a potential predictor of CDI according to our comparative analysis (OR = 1.37; 95% CI, 1.06 to 1.78) (Figure 5f). Although the number of platelets did not have a statistically significant effect (Supplementary Figure S3b), it is important to note that only three studies, apart from ours, have evaluated this parameter.

## 3. Discussion

*C. difficile* is an urgent threat to human health; therefore, understanding the epidemiology of CDI is extremely necessary to implement effective detection, control, and treatment measures. The importance of understanding both healthcare and community-related risk factors lies in helping to identify individuals who may be more susceptible to infection and mitigating the risk of CDI. In Latin America, particularly in Argentina, comprehensive epidemiological data on CDI are limited. Patients with diarrhea are not routinely tested for *C. difficile* in developing countries, and when tested, only the enzyme immunoassay (EIA) is used [29,70,71]. This could lead to an underestimation of the diagnosis and increased economic burdens on healthcare systems.

We identified that 21.29% of the patients affected by diarrhea and gastrointestinal symptoms were infected with *C. difficile*. This percentage was similar to the worldwide reported prevalence, where CDI is the underlying cause of 15 to 20% of diarrhea associated with the use of antibiotics [72]. Within the CDI+ population, 80% were infected with a toxigenic strain, which agrees with a Korean study that reported a 19.5% prevalence of infection with non-toxigenic strains [73]. In South America, molecular epidemiological surveillance is scarce (or even non-existent), with ribotypes 027, 106, 012, 046, and 014/020 reported as the most frequent [41]. In Argentina, there is only one molecular characterization that describes the ST1 strain (associated with NAP1/027) as one of the most prevalent among hospital isolates [74].

Interestingly, in our study, 43.59% of the reported cases were classified as acquired in hospital settings, and 33.33% as community-associated CDI. We also found 23.08% of the cases had a community onset but were associated with healthcare environments. The epidemiology of CDI has changed in the last two decades. The 027 strain was responsible for clinical outbreaks in the early 2000s, leading to an increase in the number of hospital-acquired cases as well as increased mortality in Europe and North America [75]. However, the incidence of this hypervirulent strain has recently decreased in part due to fluoroquinolone restrictions, prevention measures, and improvements in detection tests that have allowed better characterization of the circulating ribotypes [76,77,78,79,80]. On the other hand, an increase in strains associated with community infection (e.g., 078, 014 in Europe and 106 in the US) has been reported in recent years, with community-associated cases increasing above 40% [11,81,82,83]. Notably, our community is closely associated with the agricultural and livestock sector, with countless reservoirs for *C. difficile*.

When analyzing the number of CDI cases on an annual basis, we found that their prevalence remained around 20%, except for 2020, when the percentage of CDI+ patients doubled. During 2020, the COVID-19 pandemic broke out with the consequent preventive use of broad-spectrum antibiotics to avoid bacterial coinfections, which could be associated with an increase in the detection of CDI cases [84,85]. Nevertheless, CDI detection could have been impacted by the decrease in the number of tests, a fact that was corroborated in multiple studies carried out during the early stages of the pandemic [86,87,88]. Additionally, there could also be an underestimation of CDI cases [89] since SARS-CoV-2 frequently causes gastrointestinal symptoms similar to those of *C. difficile* [90].

Among the risk factors, advanced age is a well-established CDI risk factor that we confirmed in our meta-analysis, for which we set 69 years as the cut-off point in our study population. Elderly patients have a greater probability of receiving broad-spectrum antibiotics, being hospitalized, or staying longer in hospital settings, partly because of the presence of comorbidities [91,92]. Patients with prolonged stays in hospitals or frequently hospitalized constitute a population at risk for CDI owing to the frequent use of antibiotics and because healthcare facilities are common environments for *C. difficile* transmission [93,94]. In the studies included in the meta-analysis, previous hospitalization ranged from one month to two years, and it was globally considered a CDI-associated risk factor with the third-highest OR value. In our analysis, hospitalizations during the prior 3 months to diagnosis were considered a risk factor for the development of CDI. Furthermore, residents of health institutions for the care of elderly individuals are at greater risk [95]. In our study, approximately 15% of CDI-positive patients came from this type of establishment.

Previous episodes of CDI constitute another well-described risk factor [96,97,98]. A higher incidence (5–10 times) of initial and recurrent CDI has been observed in elderly patients, with a worse prognosis and an elevated mortality rate [95,99]. Consistently, in our study cohort, we identified previous CDI as a major risk factor, a finding strongly reinforced by our meta-analysis. In fact, this variable yielded the highest pooled effect size among all factors analyzed. Although only five studies provided this variable, the forest plot revealed a uniform trend, clearly reflected by the measure of inter-study heterogeneity, highlighting the robustness of previous CDI as a predictor of new episodes.

Previous consumption of antibiotics is directly related to dysbiosis of the microbiota, which enables the colonization of *C. difficile* and is typically considered the main risk factor for CDI [100,101]. It has also been reported that a longer duration of antibiotic therapy, as well as a greater number of antibiotics administered, increases the probability of acquiring a *C. difficile* infection [102]. The risk of acquiring CDI is estimated to be 8 to 10 times greater during antibiotic therapy, even three months after its completion, with the first month being the one with the highest risk [103]. In agreement with this, we established the consumption of antibiotics in the 3 months prior to the diagnosis of CDI as a factor that predisposes patients to CDI. Our meta-analysis also revealed previous consumption of antibiotics as a risk factor for CDI, with the second-highest OR.

There is an arduous discussion about considering PPIs as risk factors for CDI, with some reports showing that approximately 40–71.4% of hospitalized patients receive PPI therapy during hospitalization [104,105,106] and others supporting the idea that PPIs could trigger long-term adverse effects through changes in the microbiota composition [107,108]. Contrary to our local findings, where PPI use was not a significant risk factor, the pooled results of the meta-analysis demonstrated a clear association with CDI, suggesting potential differences in clinical settings.

The presence of comorbidities has been widely reported as a condition that facilitates colonization and infection by *C. difficile* [98]. Meta-analysis revealed that IBD, diabetes, leukemia or lymphoma, kidney failure, and solid cancer were the most common [109]. However, in our cohort, patients with CDI did not differ from patients without CDI regarding comorbidities, and our meta-analysis indicated that heart and kidney disease increased the risk of CDI.

We also analyzed blood and serum parameters. We detected increases in both leukocyte and platelet counts, as well as elevated levels of urea and a decrease in the number of basophils in CDI+ patients. In line with our results, some studies have highlighted that an elevated white blood cell (WBC) count is frequently observed in the context of CDI [110,111,112]. Additionally, our meta-analysis revealed leukocytosis as a risk parameter. However, few studies have explored the use of an elevated WBC count as a predictor of the CDI, and we agree with Vargas et al. [113] that the total leukocyte count alone is not a specific indicator of *C. difficile* infection.

Previous studies evaluating the platelet count during CDI episodes reported controversial results in relation to clinical symptoms [114,115,116,117,118,119,120,121,122,123]. Although in our meta-analysis no statistical significance was obtained to support thrombocytosis as a risk factor for CDI, only three studies reported this information [50,54,55]. Two of these reports revealed an elevated platelet count, which is in agreement with the results observed in our CDI+ population [50,55]. Furthermore, we have recently shown that platelets bind to *C. difficile* and promote its uptake by human macrophages via macropinocytic pathways [124]. Therefore, further studies are needed to elucidate the role of platelets during the resolution of CDI.

To the best of our knowledge, to date, there are no reports on the role of basophils in CDI. Nevertheless, the potential role of CCL-5, a basophilic recruiter chemokine, in CDI has been highlighted [125,126]. We found a low number of basophils in CDI patients.

Little research has focused on the urea concentration in the context of CDI, with high urea levels proposed as a risk factor for severe CDI [127] and elevated BUN ratios associated with complications of CDI [128], as well as with higher mortality rates [129]. In our CDI+ cohort, both the urea concentration and the BUN to creatinine ratio were increased. This is in line with our meta-analysis, where kidney diseases were found to be a predisposing factor for CDI.

Overall, we defined risk factors associated with CDI and detected modulations in different blood parameters in our study cohort. Our results highlight the use of leukocyte, platelet, and basophil counts and the BUN ratio as possible peripheral predictors in addition to the risk factors typically associated with CDI development. Finally, our study demonstrates the importance of detecting *C. difficile* as an etiological agent of hospital-acquired and community-associated infectious diarrhea in countries where testing is not standardized.

Several limitations of this study should be acknowledged. First, the relatively small number of CDI-positive cases in our cohort (~50 events) precluded the performance of multivariable analyses. Including multiple covariates in a logistic regression model under these circumstances would have increased the risk of overfitting and unstable estimates. Therefore, only univariate analyses were conducted. Future studies with larger cohorts are needed to allow for robust multivariable modeling and to confirm independent associations.

Regarding the meta-analysis, some pooled estimates showed substantial heterogeneity (I^2^ > 75%). Although this may appear concerning, such variability is not unexpected in meta-analyses of clinically and methodologically heterogeneous studies. The included reports differed in sample size, patient characteristics, country of origin, study period, type of healthcare institution, and biomarker measurement methods. Moreover, not all studies assessed the same variables. To account for between-study variability, we applied a random-effects model using restricted maximum likelihood (REML) estimation. This method assumes the presence of heterogeneity across studies and incorporates it into the analysis, yielding wider and more conservative confidence intervals. Despite these limitations, the overall consistency in the direction and magnitude of associations across studies supports the potential clinical relevance of our findings. Nonetheless, further well-designed prospective studies using standardized methodologies are warranted to validate and refine these results.

## 4. Materials and Methods

### 4.1. Human Samples and Participating Institutions

The research was carried out in accordance with the Declaration of Helsinki (2013), promulgated by the World Medical Association and approved by the UNNOBA (Universidad Nacional del Noroeste de la Provincia de Buenos Aires) Ethics Committee (COENOBA). All the samples were collected after obtaining informed consent.

Inclusion and exclusion criteria. Female and male samples from hospitalized adult patients (≥18 years) with diarrhea were collected. Diarrhea was defined as 3 or more loose watery stools in 24 h. Comorbidities were documented in the collection data chart. Exclusion criteria included pediatric patients, duplicate samples, and formed stool samples.

Unformed stools were frozen at −20 °C until use. Blood samples were obtained and analyzed on the same day as fecal sample collection. Blood cell numbers were quantified in a hematology counter. Levels of creatinine, albumin, and urea were measured using a biochemical autoanalyzer.

All samples were derived from the following healthcare centers: Hospital Interzonal General de Agudos (HIGA) Abraham Félix Piñeyro, Clínica Centro, and Clínica IMEC. These centers nucleate samples from Sanitary Region III of Buenos Aires, Argentina [130] (Supplementary Figure S1a). All participating institutions are acute-care, polyvalent hospitals that provide medium to high-complexity medical care. Samples consecutively received between 1 January 2019 and 31 December 2023 were included in this analysis.

### 4.2. Patient Characterization

We conducted a retrospective case-control study that included adult hospitalized patients (≥18 years) with diarrhea. Cases were defined as patients with CDI, while the control group consisted of CDI-negative hospitalized patients with diarrhea.

The diagnosis of CDI was based on clinical signs and symptoms in combination with the laboratory tests (Supplementary Figure S1b) applied uniformly to all patients. The presence of *C. difficile* was determined in the fecal samples via a diagnostic algorithm (Supplementary Figure S1b), as recommended by Crobach et al. in 2016 [131]. Briefly, three tests were used in a retrospective approach: Enzyme immunoassay (EIA, CoproStrip^TM^ *C. difficile* GDH + Toxin A + Toxin B (Savyon^®^ Diagnostics Ltd. (Ashdod, Israel)), Polymerase Chain Reaction from stool samples (PCR, Taq Phire Tissue Direct PCR Master Mix (Thermo Fisher (Waltham, MA, USA)), and Toxigenic culture (stool culture in CHROMAgar^TM^ *C. difficile* plates (CHROMAgar^TM^ (Paris, France)) + PCR from isolated *C. difficile* colonies).

Patients were defined as CDI+ or CDI− patients, and clinical, demographic, and blood parameters were evaluated (Table 4). Categorical variables were summarized using the number of patients in each patient group, whereas continuous variables were summarized via descriptive statistics (means and standard deviations).

CDI classifications by setting of acquisition and severity were defined according to the Infectious Diseases Society of America (IDSA) criteria [132]. Briefly, healthcare facility-onset CDI (HO-CDI) corresponds to patients who develop CDI after 72 h of hospitalization. Community-associated CDI (CA-CDI) refers to patients who have no records of hospitalizations in the last 28 days and present the infection during the first 72 h of hospitalization. Community onset associated with healthcare environments CDI (CO-HCFA CDI) corresponds to patients who present the infection in the first 72 h of the current hospitalization and who have been hospitalized during the previous 28 days.

To classify the severity of CDI, the following parameters were taken into account according to IDSA [132].

- Mild/moderate CDI: Presence of diarrhea, normal or elevated leukocyte numbers, and normal creatinine levels.
- Severe CDI: Presence of diarrhea, plus two of the following parameters: Leukocyte number greater than 15.000/mm^3^, creatinine concentration greater than 1.5 mg/dL, serum albumin less than 3 gr/dL, and/or lactate greater than 2.2 mmol/L.
- Complicated CDI: Severe CDI combined with ileus and/or hypotension and/or septic shock and/or perforation and/or requirement for ICU and/or deterioration of the sensorium and/or ascites (without another cause) and/or pseudomembranous colitis in colonoscopy, and/or imaging criteria (colon distention greater than 6 cm, thickening of the colon wall, inflammation of the pericolonic fat).

### 4.3. Meta-Analysis Design

A bibliographic search was carried out via the PubMed and Google Scholar databases in addition to the AI SciSpace tool. This study’s selection process followed the PRISMA 2020 guidelines (Tables S1 and S2). A systematic search was performed covering the period from 2000 to 2023. Initially, 7874 records were identified through searches in PubMed (n = 3100, search terms: “epidemiology” OR “cases and controls study” OR “risk factors” OR “clinical records” AND “CDI” OR “*C. difficile*”, Filters applied: Abstract, Full text, Humans, Adult: 19+ years), and Google Scholar (n = 4774, search terms: “risk factors” “case-control” “*C. difficile*” -citation). To ensure the reproducibility and integrity of the cohort, all 7874 identified records were exported and archived in Mendeley at the time of search. Following the removal of 461 records (372 duplicates and 89 records in non-English languages), 7413 titles and abstracts were screened. Of these, 7039 records were excluded using automated tools in Mendeley based on pre-defined epidemiological filters, and 305 records were manually excluded after human review. Subsequently, 69 reports were sought for retrieval and assessed for eligibility through full-text analysis. Following the exclusion of 30 reports for not strictly meeting inclusion criteria or lacking control group data, 39 studies (38 from other countries and OUR study) were included in the final meta-analysis. Three reviewers independently screened each record and report retrieved to determine whether the studies met the inclusion criteria. The reports that fulfilled the definition criteria of cases and controls were selected, and data were collected by using a customized Excel template form. The meta-analysis workflow is summarized in Figure 4a. The review protocol was not prospectively registered. However, it was retrospectively registered in the Open Science Framework database (Registration DOI: https://doi.org/10.17605/OSF.IO/X5RWD, accessed on 30 April 2026).

### 4.4. Statistical Analysis

All statistical analyses were performed comparing CDI+ and CDI− populations. For clinical, demographic, and blood parameters comparisons, the parametric *t*-test or nonparametric Mann–Whitney test for unpaired samples was used. Fisher’s exact test was used to analyze the frequency distribution of qualitative/nominal variables. Multivariable logistic regression analysis was not performed because the number of CDI-positive cases (21.29% of 249 samples) resulted in an insufficient number of outcome events to support a stable multivariable model according to the events-per-variable (EPV) criterion. The data were analyzed via GraphPad Prism 8.0.1 software (San Diego, CA, USA), and *p*-values < 0.05 were considered statistically significant.

For the meta-analysis, the “Metafor” package from RStudio (2023.06.1+524 version) was used [133]. The Odds Ratio (ORs, for categorical variables), the Standardized Mean Difference (SMDs, for continuous variables), and the inter-study heterogeneity (I^2^) were calculated. A REML (Random Effect Maximum Likelihood) approach was employed. Those models that presented a *p*-value < 0.05 were considered potential risk predictors of CDI. The methodological quality of the included studies was independently assessed using the Newcastle–Ottawa Scale (NOS). Studies were scored based on selection, comparability, and outcome/exposure criteria. Additionally, the study selection process followed the PRISMA guidelines. Potential publication bias and small-study effects were evaluated through visual inspection of funnel plots and Egger’s regression test.

## 5. Conclusions

This study emphasizes the need for optimized surveillance of the resistant pathogen *Clostridioides difficile* and the implementation of CDI diagnosis to improve public health strategies. Global concern about antimicrobial resistance increases the need to study the epidemiology of CDI and the associated risk factors. Our results revealed that *C. difficile* is the causal agent of more than 20% of cases of infectious diarrhea, with a substantial proportion of community-associated cases, consistent with global trends. We updated the available information about CDI risk factors through a meta-analysis that evaluated reports from the last 18 years, revealing that several of the risk factors detected in our study are reflected worldwide. Finally, while these findings require prospective validation, our results suggest that monitoring basophil counts, platelet levels, and BUN could provide additional insights during the clinical evaluation of CDI. Further research is needed to establish whether these parameters can reliably contribute to the diagnostic or prognostic characterization of the infection.

## Figures and Tables

**Table 1:** Countries and studies included in the meta-analysis. Selected studies to perform the meta-analysis, following PRISMA guidelines, discriminated according to country of origin.

Countries	Studies
Argentina	Lopardo, G. et al., 2015 [27] OUR study
Brazil	Lopes Cançado, G.G. et al., 2018 [33]
Canada	Lowe, D.O. et al., 2006 [34]
China	Dai, W. et al., 2020 [35] Li, Y. et al., 2016 [36] Lv, Z. et al., 2014 [37] Tang, C. et al., 2018 [38] Zhou, F.F. et al., 2014 [39]
Colombia	Carvajal, C. et al., 2017 [40] Salazar, C.L. et al., 2017 [41]
Denmark	Soes, L.M. et al., 2014 [42]
France	Le Monnier, A. et al., 2022 [43]
Hungary	Kurti, Z. et al., 2015 [44]
Iceland	Vesteinsdottir, I. et al., 2012 [45]
India	Ingle, M. et al., 2011 [46] Ingle, M. et al., 2013 [47]
Japan	Mori, N. et al., 2015 [48]
Mexico	Morfin-Otero, R. et al., 2016 [49]
Poland	Czepiel, J. et al., 2014 [50]
South Africa	Legenza, L. et al., 2018 [51] Rajabally, N. et al., 2013 [52]
South Korea	Han, S.H. et al., 2014 [53]
Taiwan	Lee, Y.C. et al., 2012 [54] Lin, C.Y. et al., 2022 [55]
Thailand	Thipmontree, W. et al., 2011 [56]
Turkey	Ergen, E.K. et al., 2009 [57]
United Kingdom	Dial, S. et al., 2005 [58] Dial, S. et al., 2008 [59] Marwick, C.A. et al., 2013 [60] Suissa, D. et al., 2012 [61] Wilcox, M.H. et al., 2008 [62]
United States	Baxter, R. et al., 2008 [63] Haddad, F.G. et al., 2019 [64] Kuntz, J.L. et al., 2011 [65] Kutty, P.K. et al., 2010 [66] Naggie, S. et al., 2011 [67] Tabak, P.Y. et al., 2015 [68] Tartof, S.Y. et al., 2015 [69]

**Table 2:** Summary of risk predictors from the meta-analysis. Odds ratios and I^2^ measure of heterogeneity for each variable evaluated in the meta-analysis.

Risk Predictor	Odd Ratios	I^2^ (Heterogeneity)
Age	1.30 ***	94.54%
Sex assigned at birth	0.91 ns	58.15%
Prior ATB consumption	3.02 ***	94.15%
Prior PPI consumption	1.43 *	93.42%
Prior hospitalization	2.69 ***	79.56%
Prior CDI	8.20 ***	00.00%
WBC (10^9^ cel/mm^3^)	1.37 *	85.63%
Platelets (10^9^ cel/mm^3^)	1.34 ns	66.15%
Comorbidities		
Diabetes *mellitus*	1.26 ns	86.02%
Heart disease	1.41 *	89.11%
Kidney disease	2.23 **	94.06%
HIV	1.00 ns	38.13%

ns = non-significant; * *p* < 0.05; ** *p* < 0.01; *** *p* < 0.001.

**Table 3:** Meta-analysis dataset. Breakdown of the global dataset included in the meta-analysis into CDI+ and CDI− patients or total cases.

		CDI+	CDI−	Total
No. of individuals		11,596	536,467	548,467
Sex assigned at birth	M	44.33%	39.98%	40.06%
	F	55.67%	60.02%	59.94%
Prior ATB consumption	With	39.95%	8.52%	9.11%
	Without	60.05%	91.48%	90.89%
Prior PPI consumption	With	29.87%	21.76%	22.18%
	Without	70.13%	78.24%	77.82%
Prior hospitalization	With	27.88%	16.80%	17.07%
	Without	72.12%	83.20%	82.93%

**Table 4:** Clinical, demographic, and blood parameters evaluated in CDI+ and CDI− patients. Categorical and continuous variables evaluated in the study cohort (CDI+ and CDI− patients).

Clinical-Demographical Parameters	Blood Parameters
Age	Leukocytes (cells/mm^3^)
Sex assigned at birth	Neutrophils (cells/mm^3^)
Previous hospitalization	Monocytes (cells/mm^3^)
Previous consumption of Antibiotics	Lymphocytes (cells/mm^3^)
Antibiotics use during hospitalization	Eosinophils (cells/mm^3^)
Presence of comorbidities	Basophils (cells/mm^3^)
Previous consumption of Proton Pump Inhibitors	Platelets (cells/mm^3^)
Previous CDI	Urea (mg/dL)
Place of hospitalization	Creatinine (mg/dL)
Need for Intensive Care Unit (ICU)	Albumin (g/dL)
Origin	
Diarrhea classification	
Shock	
Death	

## Data Availability

The clinical and original data presented in this study are available on reasonable request from the corresponding author. These data are not publicly available due to ethical restrictions and the need to protect participant confidentiality.

## Supplementary Materials

The following supporting information can be downloaded at: https://www.mdpi.com/article/10.3390/antibiotics15060528/s1, Figure S1: Sanitary Region map and diagnostic algorithm; Figure S2: Clinical data from CDI+ and CDI− patients; Figure S3: Forest plots representative of variables evaluated in the meta-analysis with non-significant results. Table S1: PRISMA_2020_abstract_checklist; Table S2: PRISMA_2020_checklist [134].
