# Supplementary material for: Epidemiology of Clostridioides difficile Infection in Argentina and Associated Risk Factors Evaluated Through a Meta-Analysis
Source: Antibiotics (Basel). 2026 May 22;15(6):528. doi: 10.3390/antibiotics15060528 (PMC13295513; doi:10.3390/antibiotics15060528)
Supplement: Supplementary file 1 [file antibiotics-15-00528-s001.zip › Supplementary Figures .pdf]

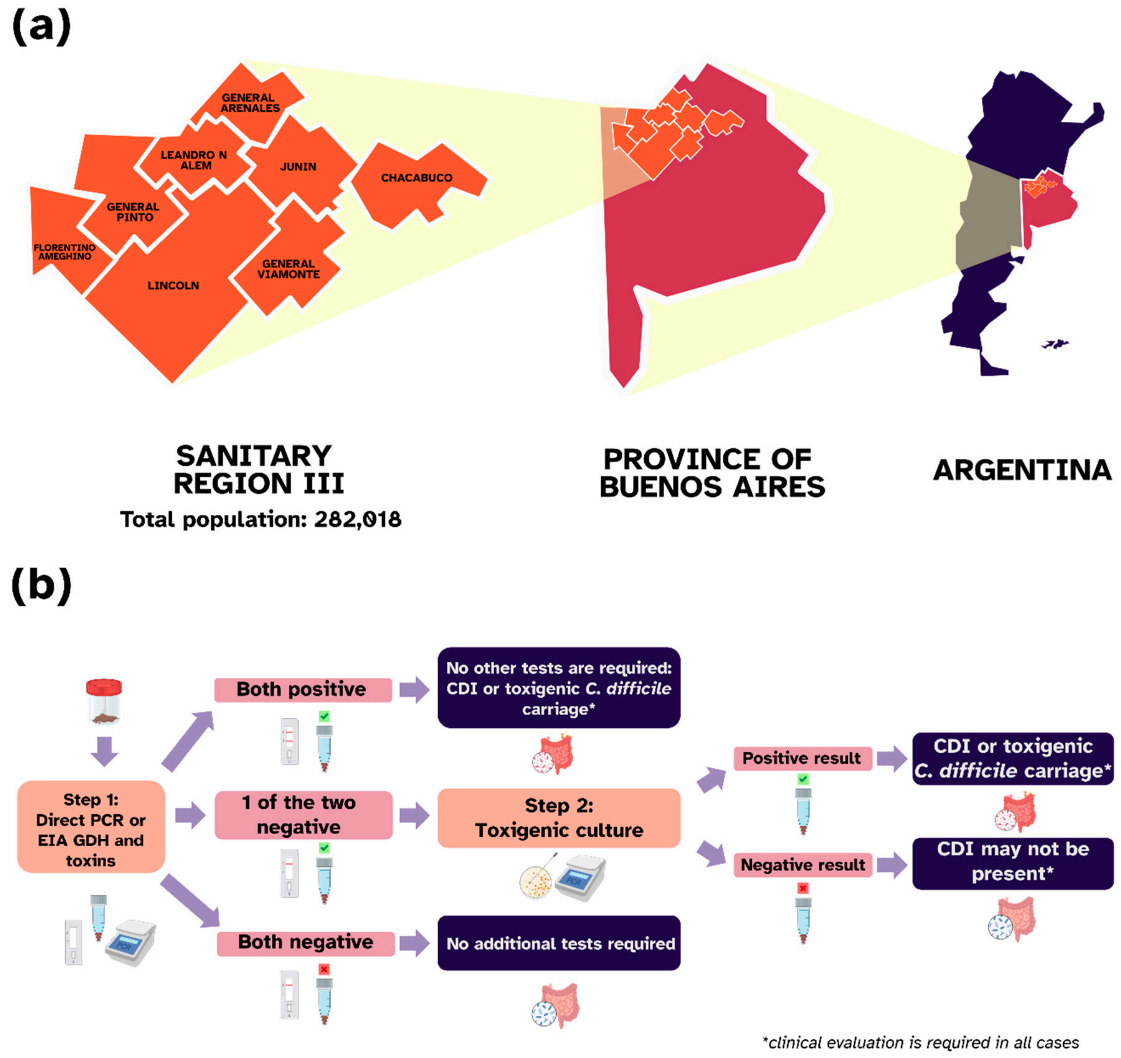

Supplementary Figure 1. (a) Sanitary Region III of Buenos Aires province, Argentina. The total population number according with the last national census (2022) is shown. (b) Diagnostic algorithm to determine *C. difficile* presence in stool samples. A cohort of 249 patients with gastrointestinal symptoms and diarrhea was evaluated. The presence of *C. difficile* within stool samples was ascertained by an algorithm that includes 3 tests (EIA, PCR, and toxigenic culture), accompanied by an exhaustive analysis of the patients' medical records. This algorithm was designed based on the recommendations of Crobach et al.
GDH, glutamate dehydrogenase from *C. difficile*

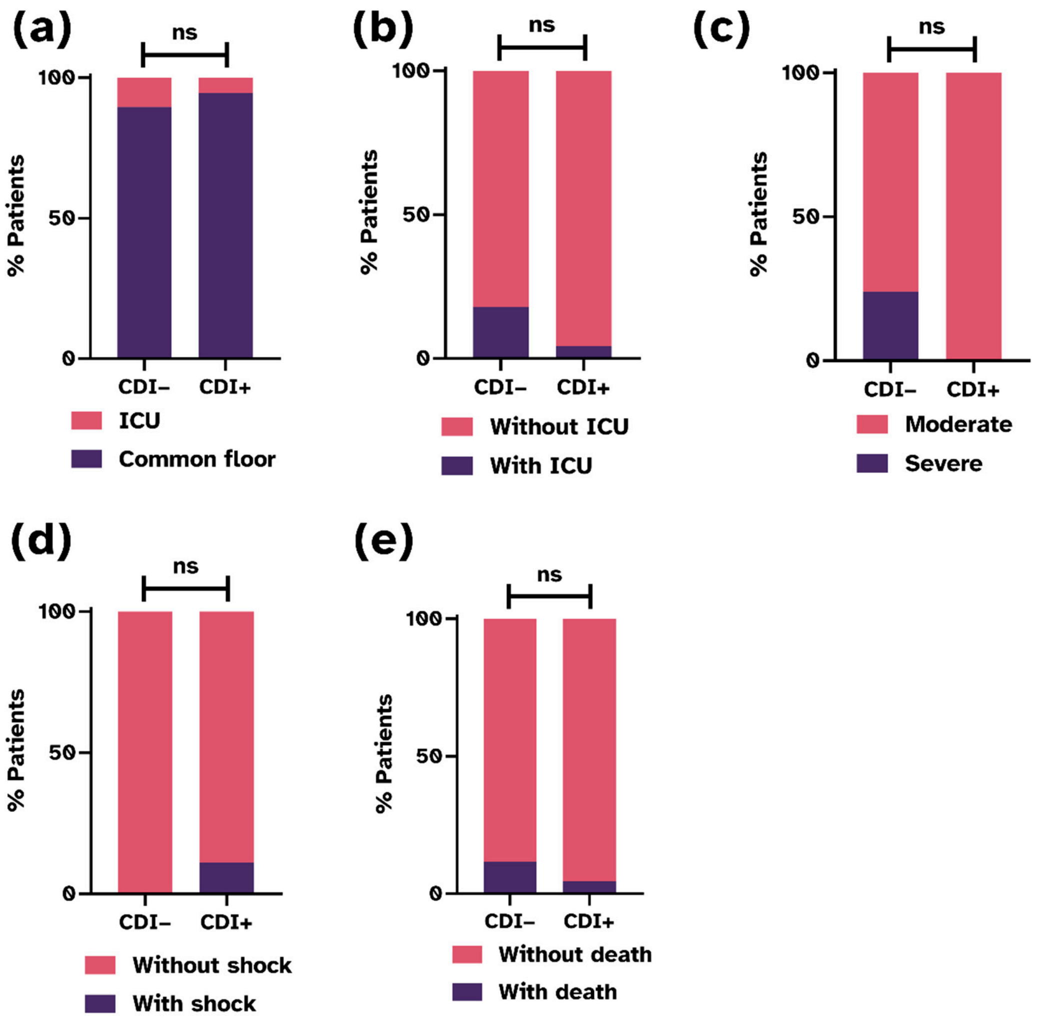

Supplementary Figure 2. Clinical data from CDI+ and CDI− patients. After the patients were classified into CDI+ or CDI− populations via the diagnostic algorithm, data from clinical records were evaluated. (a) Type of hospitalization room, (b) requirement of the ICU, (c) diarrhea classification, (d) presence of shock, (e) death.
ICU= Intensive Care Unit.
(a-e), Fisher's exact test; ns=non-significant.

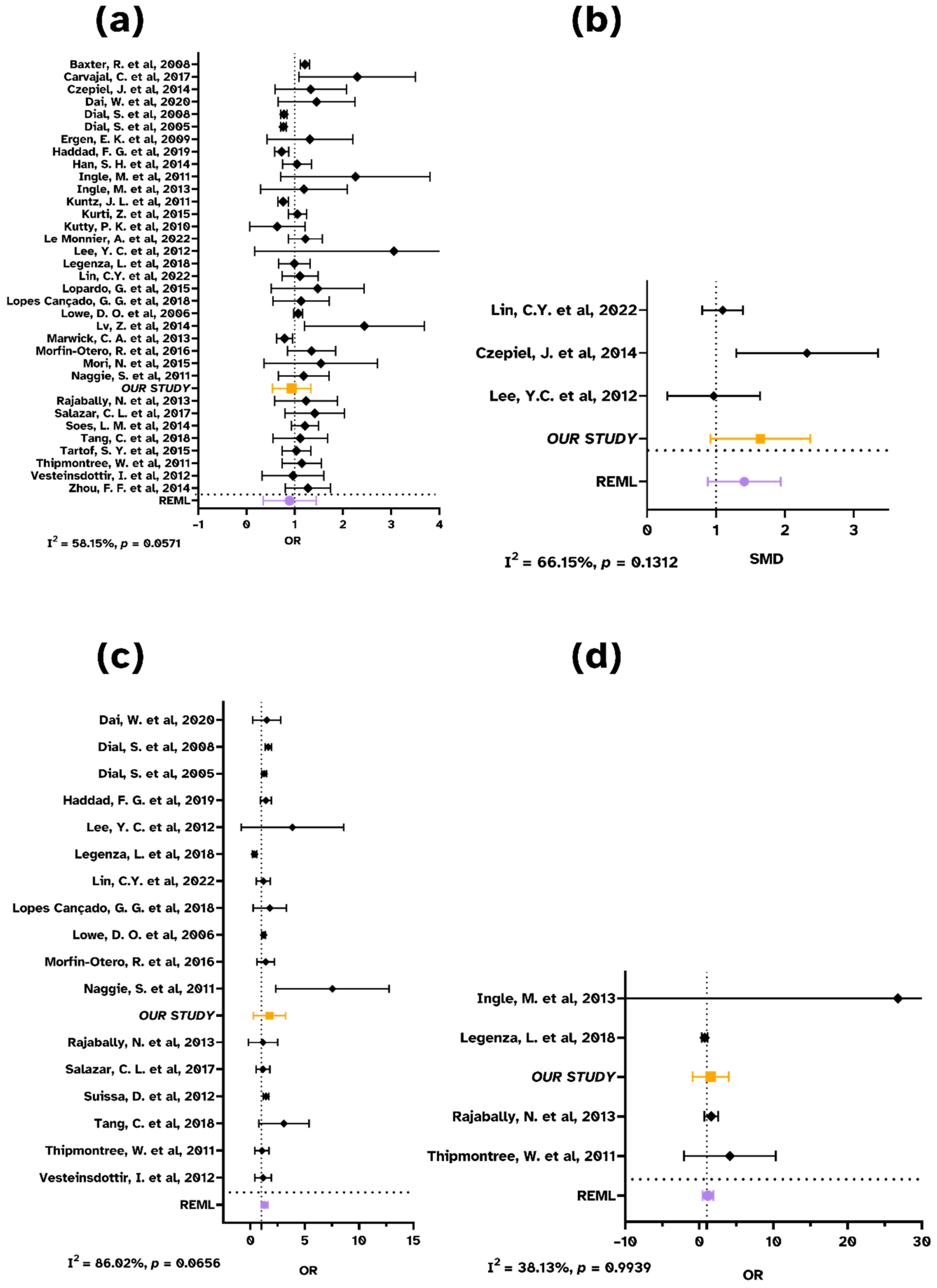

(a)
Baxter, R. et al, 2008
Carvajal, C. et al, 2017
Czepiel, J. et al, 2014
Dai, W. et al, 2020
Dial, S. et al, 2008
Dial, S. et al, 2005
Ergen, E. K. et al, 2009
Haddad, F. G. et al, 2019
Han, S. H. et al, 2014
Ingle, M. et al, 2011
Ingle, M. et al, 2013
Kuntz, J. L. et al, 2011
Kurti, Z. et al, 2015
Kutty, P. K. et al, 2010
Le Monnier, A. et al, 2022
Lee, Y. C. et al, 2012
Legenza, L. et al, 2018
Lin, C.Y. et al, 2022
Lopardo, G. et al, 2015
Lopes Cançado, G. G. et al, 2018
Lowe, D. O. et al, 2006
Lv, Z. et al, 2014
Marwick, C. A. et al, 2013
Morfin-Otero, R. et al, 2016
Mori, N. et al, 2015
Naggie, S. et al, 2011
OUR STUDY
Rajabally, N. et al, 2013
Salazar, C. L. et al, 2017
Soes, L. M. et al, 2014
Tang, C. et al, 2018
Tartof, S. Y. et al, 2015
Thipmontree, W. et al, 2011
Vesteinsdottir, I. et al, 2012
Zhou, F. F. et al, 2014
REML
-1
0
1
2
3
4
OR
I^2 = 58.15%, p = 0.0571
(b)
Lin, C.Y. et al, 2022
Czepiel, J. et al, 2014
Lee, Y.C. et al, 2012
OUR STUDY
REML
0
1
2
3
SMD
I^2 = 66.15%, p = 0.1312
(c)
Dai, W. et al, 2020
Dial, S. et al, 2008
Dial, S. et al, 2005
Haddad, F. G. et al, 2019
Lee, Y. C. et al, 2012
Legenza, L. et al, 2018
Lin, C.Y. et al, 2022
Lopes Cançado, G. G. et al, 2018
Lowe, D. O. et al, 2006
Morfin-Otero, R. et al, 2016
Naggie, S. et al, 2011
OUR STUDY
Rajabally, N. et al, 2013
Salazar, C. L. et al, 2017
Suissa, D. et al, 2012
Tang, C. et al, 2018
Thipmontree, W. et al, 2011
Vesteinsdottir, I. et al, 2012
REML
0
5
10
15
OR
I^2 = 86.02%, p = 0.0656
(d)
Ingle, M. et al, 2013
Legenza, L. et al, 2018
OUR STUDY
Rajabally, N. et al, 2013
Thipmontree, W. et al, 2011
REML
-10
0
10
20
30
OR
I^2 = 38.13%, p = 0.9939

Supplementary Figure 3. Forest plots of risk predictors. Forest plots representative of variables evaluated in the meta-analysis with non-significant results. (a) Sex assigned at birth, (b) platelet count (cells/mm^3), (c) diabetes mellitus, (d) HIV. A REML (Random Effect Maximum Likelihood, light violet circle) random effects model was applied. Models with p<0.05 and OR/SMD ± CI values less than or greater than 1, were considered potential risk predictors for CDI. The black diamonds represent the means of each of the variables in each study. The bars indicate the lower and upper confidence extremes. Our study (orange square) is mentioned as OUR STUDY. OR= odds ratio. SMD= standard media deviation. CI= confidence interval. I^2= measure of heterogeneity. (a) [27, 33 – 35, 37 – 60, 63 – 67, 69], (b) [50, 54, 55], (c) [33 – 35, 38, 41, 45, 49, 51, 52, 54 – 56, 58, 59, 61, 64, 67], (d) [47, 51, 52, 56].
