# Supplementary material for: Epidemiology of Clostridioides difficile Infection in Argentina and Associated Risk Factors Evaluated Through a Meta-Analysis
Source: Antibiotics (Basel). 2026 May 22;15(6):528. doi: 10.3390/antibiotics15060528 (PMC13295513; doi:10.3390/antibiotics15060528)
Supplement: Supplementary file 1 [file antibiotics-15-00528-s001.zip › Table S1 PRISMA_2020_abstract_checklist - revised version 3.pdf]

PRISMA 2020 for Abstracts Checklist

Section and Topic	Item #	Checklist item	Reported (Yes/No)
**TITLE**			
Title	1	Identify the report as a systematic review.	YES (Line 3)
**BACKGROUND**			
Objectives	2	Provide an explicit statement of the main objective(s) or question(s) the review addresses.	YES (Line 23 – 25)
**METHODS**			
Eligibility criteria	3	Specify the inclusion and exclusion criteria for the review.	YES, but in the manuscript (Lines 470 – 491 and Figure 4)
Information sources	4	Specify the information sources (e.g. databases, registers) used to identify studies and the date when each was last searched.	YES, but in the manuscript (Lines 470 – 491 and Figure 4)
Risk of bias	5	Specify the methods used to assess risk of bias in the included studies.	YES, but in the manuscript (Lines 487 – 491 and lines 503 - 512)
Synthesis of results	6	Specify the methods used to present and synthesise results.	YES, but in the manuscript (Table 2, Figure 5 and Supplementary figure 3, lines 503 – 512)
**RESULTS**			
Included studies	7	Give the total number of included studies and participants and summarise relevant characteristics of studies.	YES, but in the manuscript (Table 1, Lines 194 – 196)
Synthesis of results	8	Present results for main outcomes, preferably indicating the number of included studies and participants for each. If meta-analysis was done, report the summary estimate and confidence/credible interval. If comparing groups, indicate the direction of the effect (i.e. which group is favoured).	Lines 37 – 40. In the manuscript (Table 2, Table 3, Figure 5 and Supplementary figure 3; Lines 210 – 242)
**DISCUSSION**			
Limitations of evidence	9	Provide a brief summary of the limitations of the evidence included in the review (e.g. study risk of bias, inconsistency and imprecision).	YES, but in the manuscript (Lines 392 – 402)
Interpretation	10	Provide a general interpretation of the results and important implications.	YES, but in the manuscript (Lines 306 – 384)
**OTHER**			
Funding	11	Specify the primary source of funding for the review.	YES, but in the manuscript (Lines 536 – 543)
Registration	12	Provide the register name and registration number.	This systematic review was not previously registered.

PRISMA 2020 for Abstracts Checklist

From: Page MJ, McKenzie JE, Bossuyt PM, Boutron I, Hoffmann TC, Mulrow CD, et al. The PRISMA 2020 statement: an updated guideline for reporting systematic reviews. BMJ 2021;372:n71. doi: 10.1136/bmj.n71. This work is licensed under CC BY 4.0. To view a copy of this license, visit https://creativecommons.org/licenses/by/4.0/

***Lines according to the clean version of the Revised manuscript version 3.
